# Cardiac Magnetic Resonance Imaging in Alcohol-Related Myocardial Injury: A Comprehensive Review

**DOI:** 10.31083/RCM48239

**Published:** 2026-05-26

**Authors:** Lieying Xu, Wei Deng, Zhuoer Xu, Hong Ren

**Affiliations:** ^1^Department of Radiology, Sir Run Run Shaw Hospital, Zhejiang University School of Medicine, 310016 Hangzhou, Zhejiang, China

**Keywords:** magnetic resonance, multimodal imaging, heart, alcohol, myocardial injury

## Abstract

Alcoholic myocardial injury is a well-defined cardiac pathological condition associated with prolonged heavy alcohol use, characterized mainly by myocardial dilation and impaired contractile function, and may ultimately progress to heart failure. An early diagnosis and an accurate assessment of this condition are fundamental. Cardiac magnetic resonance (CMR) is a multiparametric imaging modality that offers high soft-tissue contrast resolution and can accurately quantify cardiac chamber volumes and functional parameters. CMR also enables visualization of pathological myocardial changes, including edema, inflammation, and fibrosis, through multimodal imaging techniques. This article reviews the clinical application of CMR in the evaluation of alcoholic myocardial injury, highlighting the advantages of this technique in the quantitative assessment of myocardial structural and functional abnormalities, detection of myocardial edema and fibrosis, and prognostic stratification. Overall, this study aimed to provide an evidence-based reference to support early diagnosis and timely therapeutic intervention in this condition.

## 1. Introduction

According to the World Health Organization’s Global Status Report on Alcohol and Health, in 2019, 56% of individuals aged 15 and older consumed alcohol. This consumption was responsible for 2.6 million alcohol-attributable deaths, accounting for 4.7% of all global deaths [1]. Long-term heavy alcohol consumption can lead to significant damage to various organ systems, including the nervous system, liver, and cardiovascular system [2]. Alcoholic myocardial injury refers to a range of pathological cardiac changes in the heart that result from prolonged alcohol abuse, encompassing clinical manifestations including alcoholic cardiomyopathy (ACM), cardiac dysfunction, and arrhythmias. Among these, ACM represents a typical pathological entity characterized by ventricular dilation accompanied by progressive deterioration of systolic function, ultimately progressing to irreversible heart failure [3, 4]. Toxic mechanisms of ethanol for the myocardium are multidimensional pathophysiological processes that may involve but are not limited to oxidative stress cascade reactions, mitochondrial bioenergetic metabolic dysfunction, inflammatory signaling pathway activation, calcium ion homeostasis imbalance, sympathetic nervous system dysregulation, and interstitial fibrosis progression [4, 5, 6, 7, 8, 9]. All these mechanisms result in structural remodeling and functional decompensation of the myocardium. Therefore, early diagnosis and accurate evaluation are of important clinical significance for patients’ prognosis.

Although echocardiography is widely employed due to its accessibility, ease of operation, and cost-effectiveness in evaluating ventricular volumes and systolic performance, it lacks sufficient sensitivity to detect microscopic structural alterations, including myocardial fibrosis and inflammatory infiltration. Consequently, it is limited in its ability to identify early or subclinical myocardial injury. Cardiac magnetic resonance (CMR) is considered the “gold standard” technique for evaluating cardiac function due to its high spatial resolution for soft tissues, capability for multiparametric imaging, and the absence of ionizing radiation [10]. CMR, which utilizes multimodal imaging, can effectively reveal pathological changes in the myocardium, such as myocardial edema, inflammation, and fibrosis. It can also quantify ventricular volumes and functional parameters, along with pathological characteristics. This allows for the detection of subclinical myocardial injury and provides imaging support for early diagnosis [11]. In this article, we summarize the application progress of CMR in clinical alcohol myocardial injury, focusing on the technical advantages of CMR in quantification of myocardial structure and function, pathological features, and prognosis, and provide a theoretical basis for optimizing the diagnostic strategy of this disease.

## 2. Pathophysiology of Alcohol-Related Myocardial Injury

Recent studies have clarified the complex pathophysiological process involved in alcoholic myocardial injury. Firstly, oxidative stress is a key mechanism. During heavy drinking, large amounts of reactive oxygen species are produced in the process of ethanol metabolism, inducing intracellular redox imbalance and further mediating the membrane lipid peroxidation, protein denaturation, and DNA damage, which can activate apoptotic pathways and trigger inflammatory responses, and promote fibrosis [4]. Secondly, mitochondrial dysfunction plays a significant role. Ethanol-induced oxidative stress harms mitochondrial membranes, inhibits ATP synthase activity, and significantly reduces energy metabolic efficiency in cardiomyocytes. Additionally, an imbalance in calcium homeostasis contributes to myocardial injury. Excessive intracellular calcium activates calcium-dependent protease and apoptosis-related signaling pathways, disrupting cytoskeleton structure and impairing contractile proteins, which ultimately leads to cardiac dysfunction [5]. Thirdly, immune-inflammatory responses are involved in this process. Ethanol metabolites can stimulate cardiomyocytes to enhance the secretion of pro-inflammatory factors such as tumor necrosis factor alpha (TNF-α) and interleukin 6 (IL-6) from monocyte-macrophages. This response can induce cardiomyocyte apoptosis and interstitial fibrosis via the nuclear factor kappa B (NF-κB) signaling pathway, accelerating ventricular remodeling [6, 7]. Fourthly, the excessive activation of the sympathetic nervous system is also an important compensatory mechanism related to alcoholic myocardial injury. Chronic ethanol consumption results in prolonged catecholamine release, which leads to desensitization of β-adrenergic receptors, myocardial hypertrophy, electrophysiological disturbances, and ultimately, malignant arrhythmias and heart failure [6, 8]. Finally, ethanol-induced myocardial injury prompts fibroblasts to secrete collagen, resulting in their proliferation, myocardial stiffness, and diastolic dysfunction through intertwined positive feedback loops that contribute to the net loss of cardiomyocytes, chamber dilation, and contractile reserve, thereby producing the clinical phenotype of ACM [9].

## 3. Value of CMR in the Assessment of Alcoholic Myocardial Injury

**Fig. 1.**
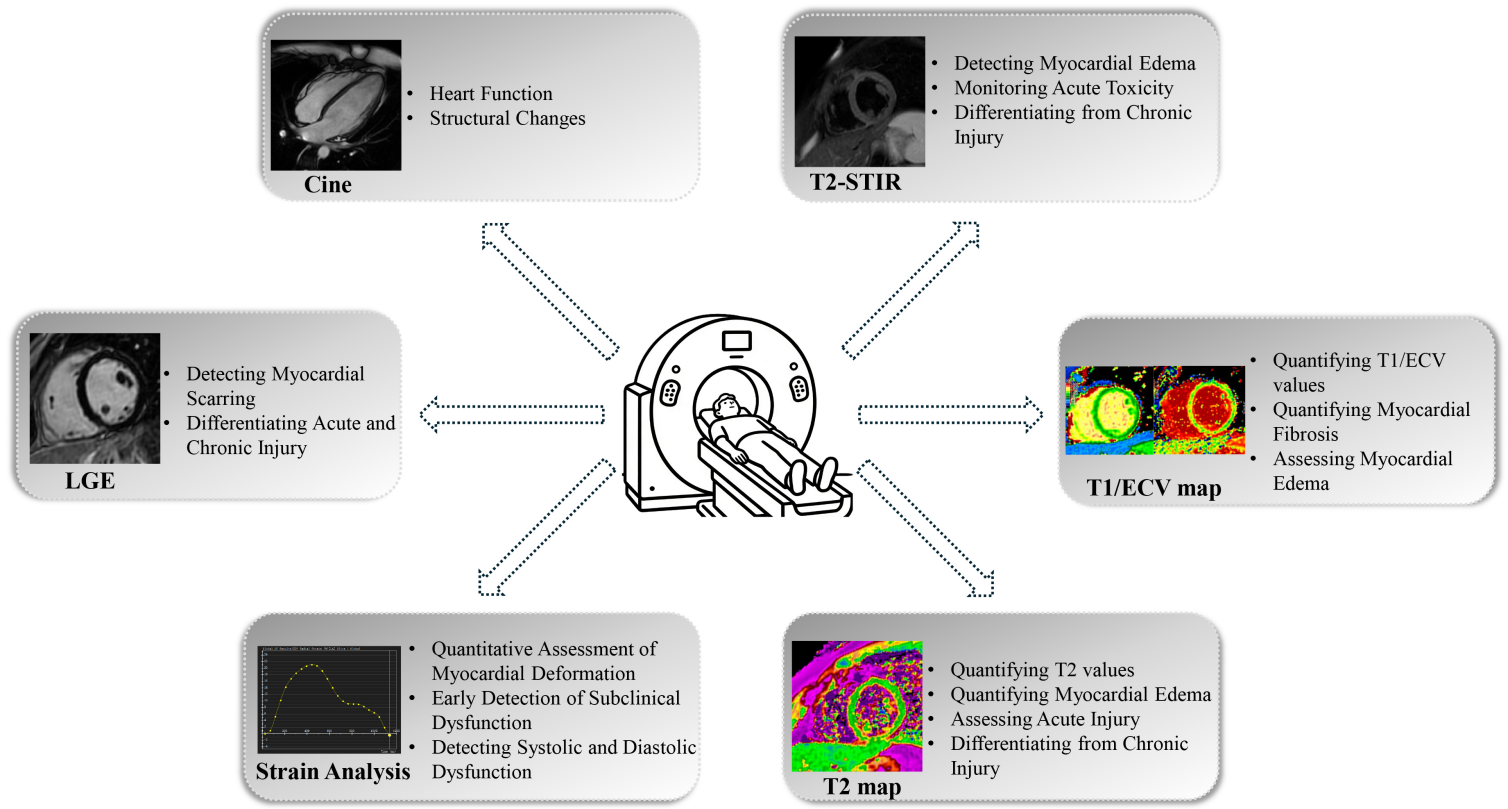
**Schematic overview of cardiac magnetic resonance (CMR) techniques for assessing alcohol-related myocardial injury**. T2-STIR, T2-short tau inversion recovery; LGE, late gadolinium enhancement; ECV, extracellular volume fraction.

Through the combination of multimodal imaging and functional quantitative assessment technology, CMR could help us assess the detailed information of myocardial structure, function, and histological features, and thus provide an accurate imaging basis for early alcoholic myocardial injury through complete evaluation and analysis of myocardial morphology, function, and histological features (Fig. 1).

### 3.1 Quantitative Assessment of Cardiac Structure and Function

CMR imaging is considered the gold standard for evaluating cardiac structure and function. It overcomes the limitation of traditional echocardiography on the geometric model assumptions and has high test-retest repeatability and inter-operator agreement. Compared to computed tomography, CMR offers advantages such as improved temporal resolution and soft tissue contrast [12]. The high temporal resolution and multiplanar imaging capabilities of CMR allow for accurate quantification of ventricular volumes, ejection fraction, and wall motion abnormalities. This imaging technique provides an objective foundation for disease staging and prognostic evaluation. Preliminary results from multiple studies indicated the possible value of CMR in the assessment of alcoholic myocardial damage. For instance, a CMR data analysis report published by Simon *et al*. [13] based on 4335 subjects from the UK Biobank found a significant positive correlation with left ventricular, right ventricular, and left atrium. This indicates that subclinical cardiac remodeling might serve as an early imaging indicator of alcohol-induced cardiovascular damage, providing robust evidence to support public health strategies for controlling alcohol consumption. The CMR study report published by Artico *et al*. [14] examined 52 patients with ACM and found that these patients often presented with reduced left ventricular ejection fraction (LVEF) of less than 50% and signs of ventricular dilatation. This study provides direct evidence of the impact of alcohol on myocardial contractile function from an imaging perspective. Tayal *et al*. [15] conducted a prospective study involving 604 patients with DCM to explore the effects of moderate-to-heavy alcohol consumption on DCM patients’ structure, function, and prognosis in those who reported drinking (n = 98) compared with those who did not drink. The study revealed that the drinking group exhibited ventricular/atrial dilatation and reduced function, as well as increased hypertrophy. However, these differences were not statistically significant after adjusting for sex. Essentially, this study indicates that moderate-to-heavy alcohol consumption is associated with cardiac structure and function abnormalities in DCM patients, and this association may have been driven by the higher proportion of males in the heavy drinking group. As mentioned above, it has been reported that differences in cardiac structure among DCM patients may be driven by lifestyle; for example, males may be more likely to consume heavy amounts of alcohol. On the other hand, emerging evidence indicates that women may demonstrate greater susceptibility to alcohol-induced myocardial injury, potentially developing functional impairment and structural remodeling at lower cumulative alcohol exposures compared with men due to differences in alcohol metabolism, body composition, and hormonal influences [16, 17]. These observations underscore the need for sex-specific considerations when defining risk thresholds and selecting candidates for CMR-based evaluation of alcohol-related myocardial injury.

CMR measurement of myocardial strain, which characterizes three-dimensional myocardial deformation patterns and can be used to characterize both regional and global systolic/diastolic function, has increasingly been used as a powerful tool for early myocardial injury because of its ability to characterize regional systolic/diastolic dysfunction [18]. Studies have shown that subclinical strain parameter changes can predict significant LVEF decreases and thus offer a time window for clinical intervention [19]. Pouliopoulos *et al*. [20] recruited 38 patients with alcohol use disorder (AUD) and acquired CMR images from these subjects. In addition to underlying left and right ventricular cardiac function impairment, these patients also demonstrated prolonged time to peak strain. Additionally, cluster analysis showed that left ventricular time to peak strain was the best predictor of AUD-related cardiomyopathy. Therefore, patients with AUD have underlying cardiac abnormalities that could be detected, and delayed peak strain might be used as a cardiovascular risk marker.

### 3.2 Assessment of Myocardial Tissue Characteristics

#### 3.2.1 Assessment of Myocardial Edema and Inflammation

Myocardial edema is one of the typical manifestations of alcoholic myocardial injury, and its early identification has important clinical value in the prevention and treatment of the disease. CMR T2-weighted imaging is an effective imaging modality for the localization of edematous areas through prolonged T2 relaxation time by taking advantage of its high sensitivity to tissue water content [21]. Zagrosek *et al*. [22, 23] performed a dynamic CMR follow-up study in 23 healthy subjects by mimicking heavy alcohol consumption. They found that the signal intensity of T2-weighted imaging increased significantly within 12 hours after alcohol intake, suggesting the appearance of myocardial edema, and returned to baseline level after one week. These findings demonstrated that even a single occasion of heavy alcohol consumption can cause transient myocardial inflammation similar to myocarditis that is detectable by MRI, and that alcohol-induced pathological changes appear to be reversible, though this requires further validation in a large population.

Myocardial inflammation is another key pathological feature of alcohol-related myocardial injury that can be evaluated by CMR. Several CMR techniques—including T2-weighted imaging, T2 mapping, native T1 mapping, and extracellular volume (ECV) quantification—allow detection of inflammatory changes by capturing increased myocardial water content, inflammatory cell infiltration, and extracellular matrix expansion [24, 25, 26]. According to the updated Lake Louise Criteria, the combined presence of T2-based (reflecting edema) and T1-based abnormalities (reflecting inflammation and extracellular expansion) significantly increases diagnostic confidence for active myocardial inflammation [27, 28]. Although direct research on CMR-detected inflammation in ACM remains limited, existing evidence, such as acute T2 elevation after heavy alcohol intake, supports the notion that alcohol can trigger transient inflammatory responses. These findings suggest that multimodal CMR may not only detect alcohol-related myocardial inflammation but also help monitor its dynamic changes over time, providing valuable insights into disease activity and progression.

#### 3.2.2 Assessment of Myocardial Fibrosis

CMR-late gadolinium enhancement (LGE) is considered the “gold standard” for characterizing myocardial scar, as it can accurately quantify focal myocardial injury and fibrosis. In recent years, the clinical significance of LGE in assessing alcoholic myocardial injury has gained recognition for differential diagnosis and prognostic stratification. A study by Artico *et al*. [14] examined LGE patterns in ACM patients compared to those with idiopathic non-ischemic dilated cardiomyopathy (iNI-DCM) patients. It was observed that in patients with ACM, LGE was most commonly localized to the mid-wall of the interventricular septum, whereas in iNI-DCM, LGE more frequently appeared in the lateral wall of the left ventricle. This finding suggests a distinctive distribution pattern that may assist in clinical differential diagnosis and reflect different underlying mechanisms. However, these preliminary results need to be validated in larger studies, preferably with genetic information, before firm conclusions can be drawn. Nevertheless, LGE detection has prognostic implications for ACM. Wang *et al*. [29] retrospectively included 62 patients with ACM to evaluate the association between alcohol-related consumption and CMR parameters (such as LGE) with prognosis, finding that continued alcohol consumption and CMR-LGE-defined myocardial scar were associated with adverse outcomes, including cardiac death, cardiac transplantation, hospitalization for heart failure, or life-threatening ventricular arrhythmias.

Traditional LGE technology has limited sensitivity in assessing diffuse myocardial fibrosis. However, T1 and ECV mapping techniques provide a promising approach for detecting early diffuse lesions through non-invasive tissue characterization [30, 31]. T1 mapping assesses changes in tissue composition by measuring native myocardial T1 values, while ECV calculates the proportion of extracellular matrix based on pre- and post-contrast T1 values and hematocrit, enabling specific identification of pathological changes such as fibrosis, edema, and inflammation. Hypothesizing that alcohol’s injury to the heart is not a focal one, researchers have explored the application of relaxation time quantification techniques in alcoholic myocardial injury. Voskoboinik *et al*. [32] found a trend of lower ECV in light-to-moderate drinkers compared to non-drinkers. However, given the relatively small sample size of 120 drinkers, these results should be interpreted with caution. Other factors, such as physiological mechanisms, such as the fat-replacement effect and the presence of comorbidities, along with the generally healthier lifestyles and higher socioeconomic status prevalent among moderate drinkers, may contribute to these findings and should be considered in future studies, socioeconomic bias [33, 34]. In contrast, there is substantial evidence linking excessive alcohol consumption to myocardial injury. Heavy alcohol intake has been shown to increase the risk of myocardial fibrosis and edema, and cardiac function gradually decreases with the increase in alcohol intake [4]. Although the T1/ECV mapping method shows sensitive detection performance for alcohol-induced myocardial microstructure changes, there are still limitations in current research: First, the observational design cannot completely exclude confounding factors; Second, alcohol intake depends on self-reporting of subjects, which may cause bias; In addition, there is no unified standard for moderate drinking. In summary, the T1/ECV mapping can provide an important basis for the early detection and dynamic changes of alcoholic myocardial injury by quantifying the myocardial tissue characteristics. In the future, we need to combine objective biomarkers in prospective studies to explore the critical threshold of the alcohol dose-response relationship and its molecular mechanism, to provide more solid evidence for clinical intervention and the formulation of public health policy.

## 4. Conclusion and Future Perspectives

As alcoholic myocardial injury is a highly complicated pathophysiological process involving multiple mechanisms in a connected manner, an early and accurate diagnosis and assessment are of great importance for clinical therapy. CMR, which has advantages in multimodal imaging and can combine structural-functional assessment and histological characterization, may play an important role in disease diagnosis and therapeutic effect evaluation. In the assessment of cardiac structural and functional assessment, in addition to accurately quantifying hemodynamic parameters such as ventricular volumes and ejection fraction and discovering subclinical systolic/diastolic dysfunction through myocardial strain analysis, MR technology can detect active myocardial edema through T2WI, localize focal fibrotic lesions through LGE technology, and use the T1/ECV mapping technique to provide objective quantitative indicators for diffuse interstitial remodeling. The combination of these techniques achieves multidimensional assessment from macroscopic to microscopic levels and provides a comprehensive imaging basis for differential diagnosis and prognostic prediction.

Despite these strengths, current CMR-based evidence on alcohol-related myocardial injury still has notable limitations. Most available studies are observational, making it difficult to control for confounding factors. Additionally, quantification of alcohol intake relies largely on self-reported data, which may introduce recall and reporting bias. The absence of a unified definition of “moderate drinking” further complicates comparisons across studies and may affect the interpretation of observed dose–response relationships. These limitations underscore the need for more rigorously designed research.

In addition, the real-world implementation of CMR-based screening faces practical constraints. Although CMR offers superior sensitivity for detecting subclinical myocardial edema, fibrosis, and subtle functional impairment, echocardiography remains the most accessible and cost-effective first-line modality for early assessment of alcohol-related myocardial injury because of its low cost and broad availability in primary care settings, especially in low-resource regions. Therefore, a pragmatic strategy would involve echocardiography for initial screening and risk stratification, with CMR reserved for individuals with inconclusive findings or those at high cumulative alcohol exposure who may benefit from detailed tissue characterization.

Moreover, since alcoholic myocardial injury is a dose-dependent condition, considering a person’s total lifetime alcohol consumption may be valuable for identifying individuals at high risk of developing ACM. Several studies [35, 36, 37, 38, 39] have suggested varying thresholds for risk, typically ranging from long-term consumption exceeding 80–90 g of ethanol per day for at least five years, or a cumulative lifetime intake exceeding several hundred kilograms, may pose a higher risk. However, there is currently no universally accepted cutoff to define high-risk individuals. Individuals with sustained heavy alcohol consumption or a significantly increased lifetime dose are more likely to exhibit subclinical myocardial alterations detectable by CMR, including ventricular dilation, reduced strain, edema, and fibrosis. Therefore, integrating an assessment of lifetime alcohol consumption into clinical evaluation may help select appropriate candidates for CMR screening, enabling earlier detection of myocardial injury before noticeable dysfunction develops.

Therefore, research using CMR to study alcoholic myocardial disease requires further development. First, large-scale multicenter studies are needed to investigate the different effects of different drinking patterns on the myocardium and to establish an early warning system based on CMR indicators. Additionally, combining CMR with other imaging and molecular biological markers may establish more comprehensive risk stratification models to predict prognosis and deliver targeted intervention strategies based on individual risk stratification. As CMR technology continues to improve, its potential application in the research of alcoholic myocardial injury is likely to expand, offering enhanced support for optimizing clinical treatment.
